# Prevalence and Association of Transfusion Transmitted Infections with ABO and Rh Blood Groups among Blood Donors at the National Blood Bank, Amman, Jordan

**DOI:** 10.3390/medicina56120701

**Published:** 2020-12-16

**Authors:** Amir M. Al Hroob, Sultan A. M. Saghir, Amer A. Almaiman, Omar S. A. Alsalahi, Abdullah S. Al-Wajeeh, Omar Y. A. Al-Shargi, Nader Al-Balagi, Ayman M. Mahmoud

**Affiliations:** 1 Department of Medical Analysis, Princess Aisha Bint Al-Hussein College of Nursing and Medical Sciences, Al-Hussein Bin Talal University, Ma’an 71111, Jordan; sultan.s.ayesh@ahu.edu.jo; 2 Department of Applied Medical Sciences, Community College of Unaizah, Qassim University, Buraydah 51431, Saudi Arabia; aamieman@qu.edu.sa; 3 Department of Medical Laboratories, Faculty of Medicine and Health Sciences, Hodeidah University, Al Hodaidah YM08, Yemen; omar_mohammed33@yahoo.com; 4 Anti-Doping Lab Qatar, Doha 27775, Qatar; a_alwajeeh@yahoo.com; 5 Department of Pharmacology, College of Pharmacy, Riyadh Elm University, Riyadh 13244, Saudi Arabia; oma12361236@gmail.com; 6 Ministry of Health, Riyadh 12233, Saudi Arabia; ph.naderbalagi@gmail.com; 7 Physiology Division, Zoology Department, Faculty of Science, Beni-Suef University, Beni-Suef 62514, Egypt

**Keywords:** blood group, Rh, blood donors, blood bank, Jordan

## Abstract

*Background and objectives:* Blood screening is considered a compulsory procedure in health care services to reduce the occurrence of transfusion transmitted infections (TTIs). This study estimated the distribution rates of ABO and Rh blood group systems, prevalence rates of TTIs among blood donors and their association with the ABO blood group and Rh system. *Materials and Methods:* A retrospective study was conducted at the national blood bank, Amman, Jordan for a period of 6 years (from January 2013 to December 2018). For TTIs analysis, about 5 mL blood sample was collected from each volunteer. A total of 365,029 persons (346,048 (94.8%) males and 18,981 (5.2%) females) donated their blood at the national blood bank, Amman, Jordan from January 2013 to December 2018. *Results:* The results revealed that O and A were the most prevalent blood groups (37.44% and 36.82%, respectively), followed by B (18.62%) and AB (7.12%). The distribution of Rh + ve and Rh − ve among blood donors showed that Rh + ve donors were more prevalent (88.73%) compared with Rh − ve (11.27%). HBsAg was the most prevalent viral infection (0.38%) followed by HCV (0.13%), syphilis (0.02%), HIV (0.006%) and the male donors were highly infected when compared with female donors. The association between ABO/Rh blood groups and TTIs infections was nonsignificant. *Conclusions:* In conclusion, low frequency rates of TTIs among blood donors were detected in the current study, but improvements are still continuously required. Low percentages of female donors need to be managed via conducting health cultural education programs.

## 1. Introduction

The transfusion of blood and its components is an important life-saving intervention that secures millions of people globally each year [1]. Over 81 million units of blood are usually donated each year. Transfusion transmitted infections (TTIs) are the most severe complications that may arise throughout blood transfusion [1]. These TTIs include human immunodeficiency virus (HIV), hepatitis B virus (HBsAg), Hepatitis C virus (HCV), and syphilis [2]. Blood or blood products that are incompetently tested or not examined represent the key cause of TTIs in both developing and developed countries [3]. The acquisition of TTIs has become a vital barrier worthy of investigation to maintain both the health and safety of human life [4,5]. In addition, TTIs have drawn considerable attention because of their persistent viremia and transient state, which could be lethal and lead to chronic and complicated life-threatening disorders [1,2]. At a global level, about 33 million are infected with HIV, 2 billion are infected with HBV and 150 million are infected with HCV [6,7]. On the other hand, syphilis, which is a disease caused by infection with *Treponema pallidum*, has been found to affect about 12 million people every year [8,9].

ABO is the main inherited blood group system responsible for the distribution of blood groups among people through the inheritance of A and B genes [1,10]. Some blood groups have been found to be able to serve as receptors and ligands for bacteria, viruses and parasites. Research has also revealed that ABO antigens can block the binding of TTI agents to polysaccharide, but cells that lack these antigens are at risk for TTIs [11,12,13]. Thus, it has been reported that there is a relationship between blood groups and some diseases, which are being affected by the distribution of blood groups [12,13]. For instance, a study conducted on blood donors in the Chinese mainland reported an association between O blood group and HBV, whereas no significant association was observed with HCV between all types of blood groups [14]. So far, a preliminary study revealed that the prevalence rates of HBV and HCV in 712 patients at dialysis units at Royal Medical Services hospitals in Jordan were 7.0% and 16.5%, respectively [15]. Another old study was conducted in 1984, which documented that the prevalence rate of HBV among blood donors at Jordan university hospital was found to be 4.4% [16].

To date, the prevalence rates of TTIs and the frequency of ABO and Rh blood groups in Jordan have not yet been studied. Therefore, this study evaluated the prevalence rates of TTIs and distribution of blood groups and Rh among blood donors at the national blood bank, Amman, Jordan from 2013 to 2018, and the possible associations between blood groups and Rh with TTIs.

## 2. Materials and Methods

### 2.1. Subjects

This is a retrospective cross-sectional study conducted over a period of 6 years (from January 2013 to December 2018) at the national blood bank, Amman, Jordan. The national blood bank provides several healthcare services to most of the hospitals and medical centers in Jordan. Ethical approval was obtained from the research ethical committee at the Faculty of Medicine, Mutah University (Ethical approval number: M/M/17-8/2019; 25 October 2012). Only healthy people with no current or previous history of infections with TTIs with ages ranging from 18 to 60 years, weight 45 kg or more, hemoglobin not less than 12.5 g/dL, and normal pulse and blood pressure can donate blood. All donors were requested to give informed consent before donation. Data were obtained from the records at the national blood bank, Amman. In the screening of blood groups, forward (cell grouping) and reverse (serum grouping) reactions were conducted to determine the blood groups of donors. The final result of grouping was confirmed with identical forward and reverse grouping.

All donors were screened for TTIs using rapid and ELISA kits purchased from BioRad (USA), following the provided instructions. Rapid immunochromatographic kits were used to screen the presence of HCV, HBsAg and venereal disease research laboratory test for syphilis (VDRL) using serum following the manufacturer’s instructions. HIV screening was done using the ELISA technique. The reactive donors were screened with nucleic acid testing (individual NAT) to preclude false positive (+ve) or false negative (−ve) cases. However, no false −ve or +ve cases were detected. The frequency rates were calculated and compared for various blood groups and TTIs. Additionally, the association of TTIs among blood donors with ABO and Rh blood groups was evaluated.

### 2.2. Statistical Analysis

Data were collected and analyzed using SPSS (version 22). ANOVA test was used to compare the frequency rates of different blood groups. A qualitative comparison to study the frequency and association between ABO/Rh blood group and TTIs was tested using Chi-square test. The differences were considered statistically significant at *p* < 0.05.

## 3. Results

### 3.1. Distribution of ABO and Rh Blood Groups

**Figure 1:**
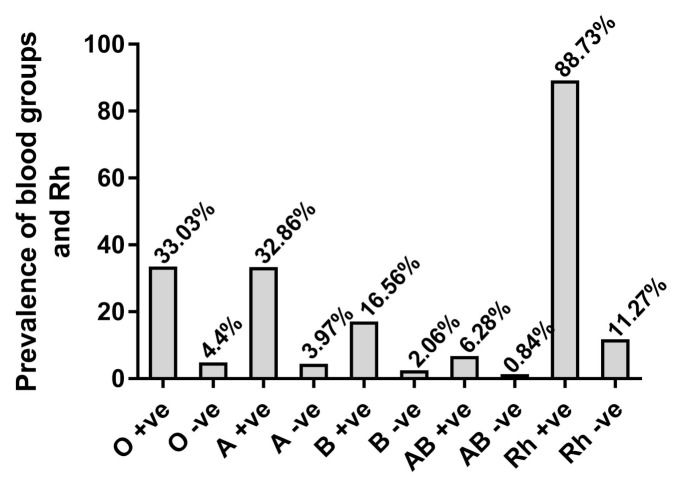
Distribution of ABO and Rh blood groups among blood donors at national blood bank, Amman, Jordan for 6 consecutive years.

In the present study, a total of 365,029 males (346,048; 94.8%) and females (18,981; 5.2%) donated their blood at the national blood bank, Amman, Jordan from January 2013 to December 2018. The average number of donors of blood is about 60,838 persons and the ages of blood donors ranged between 20–60 years with an average of 33.4 ± 8.7 years. Donors are divided into replacement donors (33%) and voluntary donors (67%). Statistically significant differences were observed between the family and volunteer donors (*p* < 0.01) and between male and female donors (*p* < 0.001). The general distribution of ABO and Rh blood groups are summarized in Figure 1. The results showed that O and A were the most prevalent blood groups (136,651; 37.44% and 134,418; 36.82%, respectively) followed by B (67,967; 18.62%) and AB (25,993; 7.12%). Detailed distributions of ABO revealed that out of 365,029 blood donors, O + ve group was the highest (33.03%), followed by A + ve (32.86%), B + ve (16.56%), AB + ve (6.28%), O − ve (4.40%), A − ve (3.97%), B − ve (2.06%), and AB − ve (0.84%). The distribution of Rh + ve and Rh − ve among blood donors is illustrated in Figure 1. Rh + ve donors were more prevalent (88.73%) when compared with Rh − ve (11.27%).

### 3.2. Prevalence of TTIs among Blood Donors

**Figure 2:**
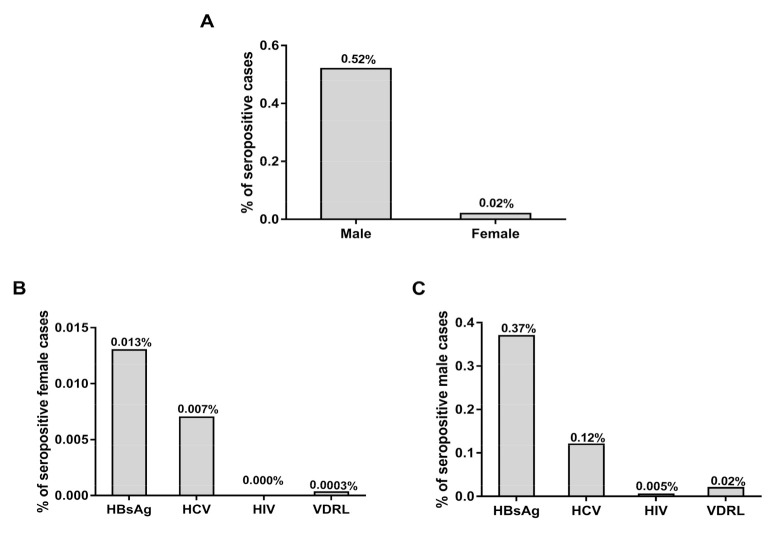
Frequency rates of transfusion transmitted infections (TTIs) among female and male donors. (**A**) Percentage of positive cases among male and female blood donors out of total donors. (**B**) Percentages of TTIs among the female donors out of total donors. (**C**) Percentages of TTIs in male blood donors out of total donors.

Figure 2 shows the percentages of TTIs seropositive cases among male and female donors out of the total number of donors. Out of the 365,029 donors, the frequency rates of TTIs in male donors (*n* = 1883) were found to be significantly higher when compared with females (*n* = 72; *p* < 0.01) (Figure 2A). The frequency rates of TTIs among female blood donors out of total donor numbers ranged from 0.013% to 0.0003% (HBsAg; 0.13%, HCV; 0.007% and VDRL; 0.0003%) and there were no HIV positive cases in female donors (Figure 2B). The frequency rates of TTIs among male blood donors out of total donor numbers ranged between 0.37–0.005% (HBsAg; 0.37%, HCV; 0.12%, HIV; 0.005% and VDRL; 0.02%) (Figure 2C).

**Figure 3:**
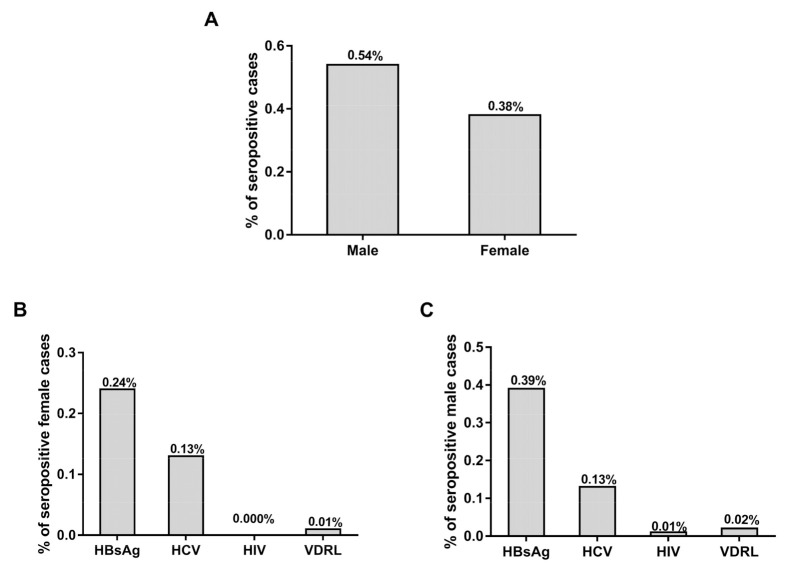
Frequency rates of TTIs among female and male blood donors. (**A**) Percentage of positive cases among male and female blood donors out of total male and female donors, respectively. (**B**) Percentages of TTIs among the female donors out of female donors. (**C**) Percentages of TTIs in male blood donors out of male donors.

The frequency rates of positive cases among female blood donors (72) out of the total number of female donors (18,981) were evaluated and compared with the frequency rates of positive cases among male blood donors (1883) out of the total number of male donors (346,048), as depicted in Figure 3. The frequency rate of TTIs positive cases among female donors out of the female donors was found to be 0.38%, while the frequency rate of TTIs positive cases male blood donors out of the male donors was found to be 0.54% (Figure 3A). The frequency rates of TTIs among female blood donors out of female donors ranged from 0.01–0.24% (HBsAg; 0.24%, HCV; 0.13% and VDRL; 0.01%) and there were no positive cases in female donors (Figure 3B). The frequency rates of TTIs among male blood donors out of male donors ranged between 0.39–0.02% (HBsAg; 0.39%, HCV; 0.13%, HIV; 0.01% and VDRL; 0.02%) (Figure 3C).

**Figure 4:**
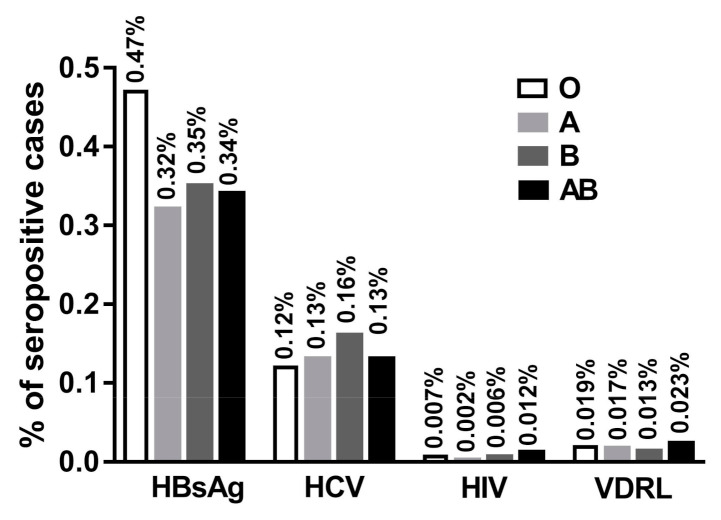
Percentages of infections with TTIs among blood donors according to the blood group of donors out of the total number of blood donors of each different group.

The percentages of positive TTIs in each blood group out of the total number of blood donors of each group were presented in Figure 4. Group O showed high percentages of HBsAg (0.47%) followed by B (0.35%), AB (0.34%) and A (0.32%) groups. For HCV, group B showed the highest percentages (0.16%) followed by A, AB and O groups. On the other hand, group AB showed the highest levels of infection with HIV (0.012%) followed by O (0.007%), B (0.006%) and A (0.002%) groups. Additionally, group AB showed the highest levels of infection with VDRL (0.023%) followed by O (0.019%), A (0.017%) and B (0.013%) groups.

The detailed distributions of the frequency rates of TTIs among male and female blood donors are illustrated in Table 1. In general, for all blood groups, HBsAg was the most prevalent viral infection (0.38%) followed by HCV (0.13%), syphilis (0.02%), HIV (0.006%), and the male donors were highly infected when compared with female donors. The frequency rate of HBsAg in O + ve donors was found to be higher (0.48%) as compared to HCV (0.13%), HIV (0.007) and VDRL (0.02) followed by group O − ve (0.37%), B + ve (0.36%) and AB + ve (0.36%). The lowest frequency rate of HBsAg infection was observed in AB − ve (0.20%). On the other hand, HCV was highly detected in B + ve (0.169%) followed by AB − ve (0.163%), A + ve (0.133%), AB + ve (0.127%), O + ve (0.125%), and A − ve (0.117%). The lowest frequency rate was detected in B − ve (0.053%). In regard to HIV infection, the highest infection was noted in AB − ve (0,33%) followed by AB + ve (0.009%), O + ve (0.007), B + ve (0.007%), O − ve (0.003%), and no HIV infections were detected in A − ve or B − ve groups. AB + ve showed the highest infections with syphilis (0.026%) followed by A − ve (0.021%) and O + ve (0.021%), A + ve (0.017%), B + ve (0.015%), and O − ve (0.006%). Group B − ve and AB − ve did not show any positive infections with syphilis.

The percentages of TTIs infection were found to be higher in RhD + ve donors compared with RhD − ve donors. The frequency rate of HbsAg and HCV were high in RhD + ve donors out of the total number of Rh + ve donors (0.39% and 0.14%) compared to RhD − ve donors out of the total number of Rh − ve donors (0.32% and 0.09%), while no differences were observed in the frequency rates of HIV and syphilis (Table 1). At the same time, statistically significant differences were detected between the number of positive cases of HBsAg and HCV in male and female donors out of the total number of male and female donors, respectively, whereas no statistically significant differences were observed in the case of HIV and syphilis.

The total numbers of blood donors every year from 2013–2018 are comparable and no statistically significant difference was observed in the total number of blood donors every year (Table 2). A gradual statistically significant decrease between the percentages of infection with HbsAg in 2013 (0.78%) as compared with 2018 (0.132%; *p* < 0.01) was observed. Simultaneously, the percentage of infection with HCV showed a statistically significant decrease in 2013 (0.18%) as compared to 2018 (0.08%; *p* < 0.05). HIV and VDRL did not show any statistically significant differences as their percentages are very low and, in some years, there was no positive case at all. No HIV + ve cases were detected in females from 2013–2018, while only one VDRL + ve case was detected in 2015. Surprisingly, the frequency rates of infection with HIV in male donors were consistently two (0.003%) for all years from 2013 to 2018. The frequency rates of syphilis in male blood donors fluctuated from year to year, as it was 0.014% in 2013 and decreased to 0.006% in 2014, then it jumped in 2015 to reach 0.031% and again dropped to 0.019% in 2016, decreasing to 0.013% in 2017, then increasing up to 0.019% in 2018. It was noted that the percentages of infection with TTIs were higher in males compared with females in all years. In the current study, no associations were observed between the TTIs and ABO and Rh blood group systems (Table 2).

## 4. Discussion

The current study is the first comprehensive study revealed the frequency rates of ABO and Rh blood group systems in male and female blood donors out of the total number of donors and out of the total number of male and female blood donors. It also aimed to assess the prevalence rates of TTIs and the possible association between TTIs with ABO and Rh blood groups. The determination of the ABO and Rh blood group systems in addition to the crossmatching between donors and recipient blood samples are considered the most important procedures, which must be performed before any blood transfusion process. Stringently following the guidelines in blood banking would help to minimize and safeguard the community from the spreading of TTIs, ensure a safe supply of blood and its products, as well as preventing the hemolytic disease of newborns [4].

This study included 365,029 donors who donated blood at national blood bank, Amman, Jordan, from 2013 to 2018. Most of them were family donors (87.60%), while the volunteer donors were approximately 14.40%. The number of male donors was higher than female ones, a finding consistent with other reports [17,18]. O and A were the most prevalent groups, followed by B and AB which is similar to the prior studies done in the Riyadh and Al-Qassim provinces (Saudi Arabia) [1,19], the American population in Cherokee, the African American population in St. Louis, the Chinese population in Hong Kong [20] and in Nigeria [21]. There was no big difference in the distribution of O and A blood groups (37.44% and 36.82%, respectively) in this study when compared with other studies in Saudi Arabia (47.45% and 26.2%) [19] and Nigeria (55.3% and 25.3%) [22] which showed significant differences in the frequency rates of O and A blood groups. Conversely, a study done by Sana and Tauseef reported that group A (28.947%) was the most prevalent followed by O (22.267%) [23], whereas other studies reported that B blood group (34.3% and 35.7%) was the most prevalent, followed by O (31.3% and 29.8%) [3,24]. The frequency rates of B and AB blood groups in this study was found to be 18.62% and 7.12%, respectively, which is lower than 35.7% and 9.7% reported by Nigam et al. [3]. In contrast, the percentage of AB blood group was higher than 3.9% reported by Alabdulmonem et al. [19]. The calculated frequency rates of Rh + ve and Rh − ve among blood donors in this study were found to be comparable with the previous results reported from Saudi Arabia [19,25]. Generally, the distribution of ABO and Rh blood group systems are differing across different populations and these differences may be attributed to many factors, such as genetic factors, and variation in sample size. On the other hand, the accumulative frequency rates of TTIs among blood donors in this study was 0.54% which is lower than 1.002% in Saudi Arabia [19], 2.3% in Yemen [26], 18.7% in New Guinea [27], 6.55% in Ethiopia [1], 37.39% in Mozambique [28], and 24% in Burkina Faso [29]. The frequency rates of positive cases of TTIs among male and female blood donors out of total number of donors were found to be 0.52% and 0.02%, respectively, which is lower than the 0.75% and 0.26% reported previously [19]. Concurrently, this study revealed that the highest positivity rates of TTIs were observed among O blood group donors (0.23%) followed by A (0.17%), B (0.098%) and AB (0.036%). The same distribution was observed in Rh + ve and Rh − ve blood donors, but the percentage of TTIs was very high in Rh + ve donors. These findings are consistent with the previous investigations conducted by Omar et al. and Mohammadali, et al. [6,12]. HBV and syphilis were found to be more prevalent in the O blood group, followed by A, B and AB, while HCV was found to be more prevalent in A followed by O, B and AB. The percentage of infection with HIV was found to be high in O blood group donors followed by B and same percentages were detected in A and AB. The findings of this study are not comparable with the prior study done by Tyagi and Tyagi and Nigam et al., where the percentages of TTIs were high among Rh − ve and A − ve blood donors when compared with other groups [3,13]. The prevalence rates of HBsAg and HCV reported here were found to be 0.38% and 0.13%, which are lower than 3.8% and 0.95% in Syria [30], 0.9% and 1.2% in Saudi Arabia [31], 1.2% and 13.6% in Egypt [32], and 2.35% and 0.79% in Yemen [26] for both HBV and HCV, respectively. At the same time, the prevalence rates of HIV and syphilis were much lower when compared with their levels in other studies [26,33], but were comparable with others [25].

Blood banks in Jordan strictly prohibit the donation from foreign individuals because they are relying on the national number to save in their database and encourage the voluntary donation through providing six months health insurance for blood donors. These procedures may be one of the factors behind the declined prevalence of TTIs in the country. The percentage of TTIs among females out of the total number of female donors was found to be highly significant when compared with the percentage of TTIs-infected females out of the total number of blood donors. Despite the fact that the frequency rate of HBsAg and HCV was very low when compared with other countries, it vastly dropped from 0.784% and 0.179% in males, and 0.024% and 0.13%, respectively, in females in 2013 to reach 0.132% and 0.08% in males and 0.010% and 0.003%, respectively, in females in 2018. The HIV and syphilis prevalence rates did not reflect much improvement as their levels were very low and no positive cases were found in female donors. In addition, the massive decrease in HBV and HCV prevalence rates from 2013 to 2018 reflected a marked improvement in Jordan’s healthcare system.

A previous study conducted by Ghazzawi et al. [15] aimed to evaluate the prevalence rates of HBsAg and HCV in hemodialysis patients at Jordanian Royal Medical Services, and reported that the frequency rates of HBsAg and HCV were 7.0% and 16.5%, respectively, which are very high compared with our findings in this study. The possible causes behind the variation of results could be the low sample size in their study (712 patients) compared with our sample size (365,029) and type of study, as they did their screening on hemodialysis patients, who are highly exposed to the infections compared with our study, which assess the rate of TTIs among healthy blood donors [15].

In the present study, male blood donors were more susceptible to TTIs when compared with the females, who could be associated with many factors, such as the behavioral and socio-cultural nature and physiology of males, and low level of health concern among males compared with females [33]. It was also noted that infected females are well diagnosed due to pre-natal care and in other settings they have more significant exposure to the tests. Another explanation documented by Goncalez et al. in 2006 and 2010 confirmed that males are more predisposed than females to be test seekers at blood centers [34,35]. The variation in the frequency rates of other TTIs could occur as a result of variations in sample size using different screening reagents and methods with different specificity and sensitivity and strict adherence to the blood transfusion guidelines [36].

The study of the association between TTTs and the ABO Rh blood group system revealed that blood group O is more susceptible to viral infection (HBsAg, HIV and syphilis) followed by blood groups A and B, whereas the A blood group is more susceptible to HCV followed by O. Despite the high prevalence rates of HBsAg among group O donors, there was no association with HBsAg, which is in line with the findings of other studies [25,37], and in contrast with others [6,38]. Likewise, other ABO and Rh blood groups did not exhibit any statistical association with the TTIs.

The current study has some limiting points that need to be addressed in future studies. We have not used advanced methods for testing TTIs, such as fourth generation ELISA and molecular testing, including nucleic acid amplification technology (NAT) for testing the viral markers. In addition, this study showed that group O was more susceptible to infection with HBsAg, HIV and VDRL, while the A blood group was more disposed to HCV infection. In addition, this study is limited by its retrospective nature. Therefore, more research to explore the relationship between antigen receptors on the O and A blood groups and the suspected infection is needed.

## 5. Conclusions

This study detected a significantly lower rate of TTIs among blood donors at the national blood bank, Amman, Jordan, but there is still a need for improvement in blood bank facilities and compliance with global blood bank guidelines. The frequency rates of O and A blood groups were much too close to each other. Group O was more susceptible to infection with HBsAg, HIV and VDRL, while the A blood group was more prone to HCV infection. The total number of Rh + ve blood donors was higher as compared to the Rh − ve blood donors and the Rh + ve blood donors showed higher percentages of seropositivity TTIs. In addition, the percentage of female blood donors was significantly lower than the percentage of male donors, and this should be addressed and improved through women’s public education about the importance and benefit of blood donations to encourage them to donate blood. Such knowledge is vital and paramount for the coordination of blood blank inventories and the delivery of transfusion services without delay to the vulnerable patients. We recommend performing more studies covering the whole country to better reflect representative results regarding the frequency rates of ABO and Rh blood group systems, as well as the prevalence rate of TTIs among communities. This would highly help in establishing a database for the blood banks in Jordan.

## Figures and Tables

**Table 1:** Distribution of TTIs among male and female blood donors according to ABO and Rh blood groups out of the total number of donors for each blood group.

Blood Group	No and % of Donors	Number and % of Male and Female	HBsAg + ve (%)	HCV + ve (%)	HIV + ve (%)	VDRL + ve (%)
O + ve	120,586	M: 114,286 (94.8)	559 (0.46)	142 (0.12)	9 (0.007)	24 (0.02)
		F: 6300 (5.2)	21 (0.02)	9 (0.007)	0 (0.00)	1 (0.000)
O − ve	16,065	M: 15,173 (94.4)	55 (0.34)	12 (0.075)	1 (0.006)	1 (0.006)
		F: 892 (5.6)	5 (0.03)	1 (0.006)	0 (0.00)	0 (0.00)
A + ve	119,941	M: 113,888 (95)	372 (0.31)	151 (0.13)	3 (0.003)	20 (0.02)
		F: 6053 (5)	8 (0.01)	8 (0.01)	0 (0.00)	0 (0.00)
A − ve	14,477	M: 13,655 (94.3)	43 (0.30)	17 (0.12)	0 (0.00)	3 (0.0.02)
		F: 822 (5.7)	1 (0.007)	0 (0.00)	0 (0.00)	0 (0.00)
B + ve	60,450	M: 57,385 (94.9)	211 (0.35)	98 (0.16)	4 (0.007)	9 (0.015)
		F: 3065 (5.1)	7 (0.11)	4 (0.007)	0 (0.00)	0 (0.00)
B − ve	7517	M: 7095 (94.4)	20 (0.27)	4 (0.05)	0 (0.00)	0 (0.00)
		F: 422 (5.6)	1 (0.013)	0 (0.00)	0 (0.00)	0 (0.00)
AB + ve	22,920	M: 21,714 (94.70)	79 (0.34)	27 (0.118)	2 (0.009)	6 (0.03)
		F: 1206 (5.3)	3 (0.01)	2 (0.009)	0 (0.00)	0 (0.00)
AB − ve	3073	M: 2852 (92.8)	6 (0.20)	4 (0.13)	1 (0.32)	0 (0.00)
		F: 221 (7.2)	0 (0.00)	1 (0.32)	0 (0.00)	0 (0.00)
Total	365,029	M: 346,048(94.8)	M:1345(0.37)	M: 455(1.25)	M: 19(0.005)	M: 63(0.02)
		F: 18,981(5.2)	F: 46(0.013)	F: 25(0.007)	F: 0(0.00)	F: 1(0.0003)

**Table 2:** Numbers and percentages of TTIs among male and female blood donors from 2013–2018.

Year	No of Donors	HBsAg	HCV	HIV	VDRL
		N (%)	N (%)	N (%)	N (%)
2013	62,078	M: 487 (0.784)	M: 111 (0.179)	M: 2 (0.003)	M: 9 (0.014)
		F: 15 (0.024)	F: 8 (0.013)	F: 0 (0.00)	F: 0 (0.00)
2014	64,394	M: 270 (0.419)	M: 71 (0.110)	M: 2 (0.003)	M: 4 (0.006)
		F: 8 (0.012)	F: 1 (0.002)	F: 0 (0.00)	F: 0 (0.00)
2015	60,677	M: 245 (0.404)	M: 99 (0.163)	M: 0 (0.00)	M: 19 (0.031)
		F: 8 (0.013)	F: 6 (0.010)	F: 0 (0.00)	F: 1 (0.002)
2016	57,750	M: 157 (0.272)	M: 74 (0.128)	M: 2 (0.003)	M: 11 (0.019)
		F: 5 (0.009)	F: 1 (0.002)	F: 0 (0.00)	F: 0 (0.00)
2017	59,680	M: 106 (0.178)	M: 52 (0.087)	M: 2 (0.003)	M: 8 (0.013)
		F: 4 (0.007)	F: 7 (0.012)	F: 0 (0.00)	F: 0 (0.00)
2018	60,450	M: 80 (0.132)	M: 48 (0.08)	M: 2 (0.003)	M: 12 (0.019)
		F: 6 (0.010)	F: 2 (0.003)	F: 0 (0.00)	F: 0 (0.00)

## References

[1] Mohammed Y., Bekele A. (2016). Seroprevalence of transfusion transmitted infection among blood donors at Jijiga blood bank, eastern Ethiopia: Retrospective 4 years study. BMC Res. Notes.

[2] Shah M., Shah S., Gajjar M., Bhatnagar N., Soni S., Patel V. (2016). Prevalence of HIV-I/II, HCV, HBsAg& Syphilis in Blood Donors of Western Region in India. Natl. J. Integr. Res. Med..

[3] Nigam J.S., Singh S., Kaur V., Giri S., Kaushal R.P. (2014). The prevalence of transfusion transmitted infections in ABO blood groups and Rh type system. Hematol. Rep..

[4] Li C., Xiao X., Yin H., He M., Li J., Dai Y., Fu Y., Ge J., Yang Y., Luan Y. (2012). Prevalence and prevalence trends of transfusion transmissible infections among blood donors at four Chinese regional blood centers between 2000 and 2010. J. Transl. Med..

[5] Gottlieb M.S., Schroff R., Schanker H.M., Weisman J.D., Fan P.T., Wolf R.A., Saxon A. (1981). Pneumocystis carinii pneumonia and mucosal candidiasis in previously healthy homosexual men: Evidence of a new acquired cellular immunodeficiency. N. Engl. J. Med..

[6] Mohammadali F., Pourfathollah A. (2014). Association of abo and Rh blood groups to blood-borne infections among blood donors in Tehran-Iran. Iran. J. Public Health.

[7] Branch D.R. (2010). Blood groups and susceptibility to virus infection: New developments. Curr. Opin. Hematol..

[8] Kokki I., Smith D., Simmonds P., Ramalingam S., Wellington L., Willocks L., Johannessen I., Harvala H. (2016). Hepatitis e virus is the leading cause of acute viral hepatitis in Lothian, Scotland. New Microbes. New Infect..

[9] WHO (2001). Global Prevalence and Incidence of Selected Curable Sexually Transmitted Infections: Overview and Estimates.

[10] Panda M., Kar K. (2008). HIV, hepatitis B and C infection status of the blood donors in a blood bank of a tertiary Health Care Centre of Orissa. Indian J. Public Health.

[11] Agarwal N. (2014). Response rate of blood donors in the Uttarakhand region of India after notification of reactive test results on their blood samples. Blood Transfus..

[12] Omar A. (2012). The infection with HBV and HCV and their relationship to abo blood group among blood donors. J. Fac. Med..

[13] Tyagi S., Tyagi A. (2013). Possible correlation of transfusion transmitted diseases with Rh type and abo blood group system. J. Clin. Diagn. Res. JCDR.

[14] Gao X., Cui Q., Shi X., Su J., Peng Z., Chen X., Lei N., Ding K., Wang L., Yu R. (2011). Prevalence and trend of hepatitis C virus infection among blood donors in Chinese mainland: A systematic review and meta-analysis. BMC Infect. Dis..

[15] Ghazzawi I., Yassin M., Alshebly H., Sheyyab S., Alqudah B., Alwahadni N. (2015). Prevalence of hepatitis B and C viruses in hemodialysis patients at JRMS. J. R. Med. Serv..

[16] Awidi A., Tarawneh M., El-Khateeb M., Hijazi S., Shahrouri M. (1984). Incidence of hepatitis b antigen among Jordanian volunteer blood donors. Public Health.

[17] El-Hazmi M.M. (2004). Prevalence of HBV, HCV, HIV-1, 2 and HTLV-I/II infections among blood donors in a teaching hospital in the central region of Saudi Arabia. Saudi Med. J..

[18] Giri P.A., Yadav S., Parhar G.S., Phalke D.B. (2011). Frequency of abo and rhesus blood groups: A study from a rural tertiary care teaching hospital in India. Int. J. Biol. Med. Res..

[19] Alabdulmonem W., Shariq A., Alqossayir F., AbaAlkhail F.M., Al-Musallam A.Y., Alzaaqi F.O., Aloqla A.A., Alodhaylah S.A., Alsugayyir A.H., Aldoubiab R.K. (2020). Sero-prevalence abo and Rh blood groups and their associated transfusion-transmissible infections among blood donors in the central region of Saudi Arabia. J. Infect. Public Health.

[20] Mourant A.E., Kipec A.C. (1976). Domaniewska-Sobczak, Kazimiera. The Distribution of Human Blood Groups.

[21] Egesie U., Egesie O., Usar I., Johnbull T. (2008). Distribution of abo, rhesus blood groups and haemoglobin electrophoresis among the undergraduate students of Niger delta university Nigeria. Niger. J. Physiol. Sci..

[22] Adeyemo O.A., Soboyejo O.B. (2006). Frequency distribution 0f ABO, RH blood groups and blood genotypes among the cell biology and genetics students of university of Lagos, Nigeria. Afr. J. Biotechnol..

[23] Ullah S., Ahmad T. (2015). Distribution of abo and Rh (D) blood groups in the population of District Dir Lower, Khyber Pakhtunkhwa Pakistan. World Appl. Sci. J..

[24] Karim S., Hoque M.M., Hoque E., Begum H.A., Rahman S.M., Shah T.A., Hossain S.Z. (2015). The distribution of abo and rhesus blood groups among blood donor attending transfusion medicine department of Dhaka Medical College Hospital in 2014. J. Dhaka Med. Coll..

[25] Almaiman A.A., Almaiman S.H. (2018). Evaluation of blood donors and transfusion transmitted infections and their association with abo and Rh blood groups in Unaizah, Saudi Arabia: A retrospective study. Int. J. Med. Res. Health Sci..

[26] Saghir S.A.M., Al–Hassan F.M., Alsalahi O.S.A., Alhariry A.-A., Baqir H.S. (2012). Frequencies of HBV, HCV, HIV, and syphilis markers among blood donors: A hospital-based study in Hodeidah, Yemen. Trop. J. Pharm. Res..

[27] Xie D.-D., Li J., Chen J.-T., Eyi U.M., Matesa R.A., Obono M.M.O., Ehapo C.S., Yang L.-Y., Yang H., Yang H.-T. (2015). Seroprevalence of human immunodeficiency virus, hepatitis B virus, hepatitis C virus, and Treponema Pallidum infections among blood donors on Bioko Island, Equatorial Guinea. PLoS ONE.

[28] Stokx J., Gillet P., De Weggheleire A., Casas E.C., Maendaenda R., Beulane A.J., Jani I.V., Kidane S., Mosse C.D., Jacobs J. (2011). Seroprevalence of transfusion-transmissible infections and evaluation of the pre-donation screening performance at the Provincial Hospital of Tete, Mozambique. BMC Infect. Dis..

[29] Nagalo B.M., Bisseye C., Sanou M., Kienou K., Nebié Y.K., Kiba A., Dahourou H., Ouattara S., Nikiema J.B., Moret R. (2012). Seroprevalence and incidence of transfusion-transmitted infectious diseases among blood donors from regional Blood Transfusion Centres in Burkina Faso, West Africa. Trop. Med. Int. Health.

[30] Othman B.M., Monem M.S.F.S. (2002). Prostitutes in Damascus, Syria. Saudi Med. J..

[31] Al Omar A.S., Zuebi F.E. (1996). Disease markers in blood donors at King Fahad Hospital, al Baha. Ann. Saudi Med..

[32] Darwish M.A., Raouf T.A., Rushdy P., Constantine N.T., Rao M.R., Edelman R. (1993). Risk factors associated with a high Seroprevalence of hepatitis c virus infection in Egyptian blood donors. Am. J. Trop. Med. Hyg..

[33] Sabino E.C., Gonçalez T.T., Carneiro-Proietti A.B., Sarr M., Ferreira J.E., Sampaio D.A., Salles N.A., Wright D.J., Custer B., Busch M. (2012). HIV prevalence, incidence and residual risk of transmission by transfusions at reds-ii blood centers in brazil. Transfusion.

[34] Goncalez T., Sabino E., Murphy E., Chen S., Chamone D., McFarland W. (2006). Human immunodeficiency virus test-seeking motivation in blood donors, Sao Paulo, Brazil. Vox Sang..

[35] Goncalez T., Sabino E., Sales N., Chen Y.H., Chamone D., Busch M., Murphy E., Custer B., McFarland W. (2010). Human immunodeficiency virus test-seeking blood donors in a large blood bank in São Paulo, Brazil. Transfusion.

[36] Glynn S.A., Kleinman S.H., Schreiber G.B., Busch M.P., Wright D.J., Smith J.W., Nass C.C., Williams A.E. (2000). Trends in incidence and prevalence of major transfusion-transmissible viral infections in us blood donors, 1991 to 1996. JAMA.

[37] Behal R., Jain R., Behal K.K., Bhagoliwal A., Aggarwal N., Dhole T. (2008). Seroprevalence and risk factors for hepatitis b virus infection among general population in northern India. Arq. Gastroenterol..

[38] Dirisu J.O., Alli T.O., Adegoke A.O., Osazuwa F. (2011). A survey of prevalence of serum antibodies to human immunodeficiency deficiency virus (HIV), hepatitis B virus (HBV) and hepatitis C virus (HCV) among blood donors. North Am. J. Med. Sci..

